# Development of a liquid phase radioimmunoassay for the measurement of serum ferritin levels for the detection of Covid-19 in patients

**DOI:** 10.1007/s10967-022-08208-1

**Published:** 2022-02-05

**Authors:** Emil Michael Riad, Manal Asem Emam, Nahed Hassan Ebeid, Ahmed Sami El-bayoumy, Khaled Mohamed Sallam, Nagy Lahzy Mehany, Shadia Abdel-Hamid Fathy

**Affiliations:** 1 Department of Labeled Compounds, Hot labs Centre, Atomic Energy Authority, Cairo, Egypt; 2 Department of Biochemistry, Faculty of Science, Ain Shams University, Cairo, Egypt

**Keywords:** Ferritin, Covid-19, Radioimmunoassay, Labelling, I^125^, Tracer

## Abstract

The aim of this study was the development and analytically validation of a radioimmunoassay system for the measurement of the serum ferritin concentration as one of the laboratory biomarkers for infection by Covid-19. The main components of the system were prepared in our laboratories. The first component ferritin was extracted and purified from human spleen with high purity. The second component was the 125I-labelled ferritin tracer, prepared using Chloramine-T method. Furthermore anti-ferritin antibodies and ferritin standards were provided. The developed system is sensitive, precise, reproducible and.

can be translated into a kit formulation suitable for measuring serum ferritin for the detection of Covid-19 in patients at low costs and high efficiency.

## Introduction

COVID-19 (Coronavirus disease-2019) is an emerging infectious disease that has been stated as a worldwide public health emergency by the World Health Organization (WHO). Since the start of the pandemic in Wuhan, China, over 177 million cumulative cases and about 3.9 million deaths globally worldwide was recorded [1]. COVID-19 has a mild influenza-like infection or may be asymptomatic, a small percentage of patients progress acute respiratory distress syndrome (ARDS), severe pneumonia, multi-organ failure, and can even die [2].

Patients with COVID-19 show hyperinflammation and concomitant biomarkers which consist of elevated serum ferritin, C—reactive protein (CRP), procalcitonin (PCT), D-dimer and this may be helpful for risk stratification. [3–6]

The amount of iron stored in the body correlates with serum ferritin levels in both healthy and sick people. Unlike serum iron, serum ferritin concentration does not fluctuate from day to day. Because of its relatively high stability and solubility, as well as its direct proportionality to body iron stores in normal persons, serum ferritin is the most commonly used indicator of total body iron storage [7, 8].

Serum ferritin is one of many blood tests used to accurately diagnose and treat a wide range of diseases, and it is likely the most effective marker in most populations [9].

Radioimmunoassay technique for serum ferritin detection depends on several components which was locally prepared to decrease the cost and increase availability of the kit; starting with extraction and purification of ferritin from human spleen to use it as an antigen used for preparation of ^125^I-Ferritin tracer, induce production of ferritin antibodies with high specificity and affinity for ferritin antigen and also to prepare wide range of ferritin standards. This report use double antibody liquid phase technique to prepare the system and separate bound from free ferritin antigen [10, 11].

The aim of this study was to develop ferritin radioimmunoassay system to facilitate detection of serum ferritin level using locally prepared components which will provide a low cost, long term uniform batches, efficient specifications for medical diagnostic purposes, as well as avoiding problems resulted from irregular supply and deterioration of the sensitive biodegradable materials during transportation from the original manufacturing countries. The prepared kit serves to cover the increased demand by physicians for ferritin test which is considered a useful laborartory biomarker for infection by Covid-19.

## Experimental

### Reagents

Phosphate buffer 0.5 M, pH 7.4; Phosphate buffer saline (PBS) 0.05 M, pH 7.4; assay buffer: 0.05 M PBS pH7.4 containing 1 mg /ml bovine serum albumin; Chloramine-T (Ch-T); Sodium metabisulphite (MBS); Normal (inactivated) rabbit serum (NRS); sheep anti-rabbit serum (2nd ab) locally produced; PEG-8000; Na^125^I (3700 MBq/ml) carrier and reductant free (Institute of Isotopes, Budapest, Hungary). All other chemicals used in this report were provided from Sigma Chemical Co. with high quality and purity.

#### Ferritin extraction and purification

Ferritin was extracted from human spleen obtained at autopsy from the Department of Pathology of Zagazig University Hospital, The tissue was frozen and stored at -10 °C till used. The extraction and purification steps applied based on the methods of Suryakala et al. [12] Cetinkaya et al. [13] and Page et al. [14] as following:

##### Homogenation

The tissue was cut into small pieces and homogenized for 10 min at 4 °C in Waring blender after adding 1.5 fold of distilled water.

##### Heat denaturation

The homogenate was heated rapidly to 70 °C in a water bath and maintained at 70–75 °C for 10 min with continuous stirring, after cooling, the homogenate was centrifuged for 30 min at 5000 rpm and remove the coagulated protein precipitated. The supernatant was then filtered through whatman filter paper no. 1 using Buchner funnel and suction to remove traces of low density coagulum.

##### Ammonium sulphate precipitation

Crude ferritin was precipitated by half saturated ammonium sulphate added slowly with constant stirring. Adjust pH at 5.2 while adding ammonium sulphate. The solution was left overnight at 4 °C then centrifuge at 2000 rpm for 30 min the supernatant was discarded and the insoluble fraction which contained the ferritin fraction was dissolved in a minimal volume of distilled water. The precipitation procedure, using 50% ammonium sulphate, was repeated twice more. Finally, the final precipitate was dissolved in the smallest possible volume of distilled water and dialyzed exhaustively overnight at 4 °C against distilled water.

##### Gel filtration on Sephacryl S-300

Six ml of the crude ferritin preparation was applied to a 2.6 × 100 cm column filled with Sephacryl S-300 equilibrated with Tris–HCl buffer, 0.05 M, pH 7.1; containing 0.1 M NaCl, at a rate of passage of 40 ml/h, which was maintained with the aid of peristaltic pump. The protein in the eluate was recorded from its absorption at 280 mm with the aid of the UV–Visible spectrophotometer. The ferritin fraction was easily identified from its characteristic orange-brown coloration. This step was repeated for the fractions containing ferritin with high concentration for more purification.

After each step the protein and ferritin contents were measured using commercial colorimetric kit (Spinreact) and enzyme immunoassay kit (Monobind) respectively. The purity and recovery percent were calculated mathematically and recorded.

#### Preparation of ^125^I- ferritin tracer

Labelling of ferritin with 125I takes place using Hunter and Greenwood method (1962) [15] with slight modification. To obtain a high radiochemical yield and a good radiochemical purity all parameters such as effect of reaction time, pH, concentration of oxidizing agent (Chloramine-T) and concentration of substrate (ferritin) were studied. Labelling process starts by dissolving 10 μg ferritin in 10 μl PBS 0.05 M pH 7.4 in eppendorf, followed by addition of 50 μl of phosphate buffer saline (0.5 M, pH 7.4), and 10 µl of Na125I of 1 mCi (37 MBq) activity. The reaction was started by addition of 10 μl chloramine-T (2 mg Ch-T dissolved in 1 ml of PBS 0.5 M pH 7.4) as oxidizing agent. The reaction mixture was gently vortexed for 30 s. at room temperature and quenched by the addition of 50 µl of sodium metabisulphite (2 mg MBS dissolved in 1 ml dist. Water) followed by addition of 5 μl of 0.1 M KI as a carrier. The labeling yield and specific activity of the produced tracer was calculated after purification using Sephadex G-25 column at flow rate 0.5 ml/min. The radiochemical purity of the tracer was detected using paper electrophoresis.

### Optimization of factors affecting the iodination process

*Effect of reaction time:* The iodination technique was studied with different reaction time ranged from 20 to 600 s.

*Effect of pH:* The iodination technique was studied using buffer at different pH values ranged from 4 to 10.

*Effect of oxidizing agent (chloramine-T) concentration:* The iodination technique was carried out using different concentrations of the oxidizing agent (Ch-T) ranged from 5 to 40 μg.

*Effect of substrate (ferritin) concentration: *The iodination process was carried out using different concentrations of the substrate (ferritin) ranged from 2.5 to 20 μg.

#### Production of anti-ferritin polyclonal antibodies

Production of ferritin polyclonal antibody was carried out through immunization of four male mature white New- Zealand rabbits weighing 2–3 kg (R1-R4) with purified ferritin antigen as immunogen according to Barnett et al. [16] and Goldie and Thomas [17]. Five injections were administered, one primary and four booster injections, at four weeks intervals. For primary injection, each rabbit received 400 µg of immunogen in 1 ml emulsion prepared by mixing with complete Freund’s adjuvant. For booster injections, each rabbit received 200 µg of the immunogen in 1 ml emulsion prepared by mixing with incomplete Freund’s adjuvant according to Zola H. [18] and Pillai and Bhandarkar [19]. Blood samples were collected 3 weeks after each injection and antibody titre was assessed by measuring the final dilution necessary to bind 50% of labelled ferritin used in the assay.

#### Preparation of ferritin standards

A stock standard of ferritin at a concentration of 10 mg/1 was prepared in assay buffer and suitable portions were frozen at− 20 °C until needed. On the day of assay a portion was removed and, after thawing, a series of working standards was prepared by dilution in the same buffer to cover the range 0–1000 ng/ml [20].

### Optimization of the liquid-phase RIA system

The assay range was set at 0–1000 ng/ml. Standard curves were set up and studied at different radio-activities of the tracer, different temperatures (4, 25 and 37 °C) and different incubation time ranged from (1 to 24 h). The optimal concentrations of the reagents and the assay reaction parameters were chosen to give a standard curve with a desirable slope and minimum imprecision.

## Results and discussion

Ferritin extraction and purification was considered the backbone for the prepared system because it produces the main component which used for preparation of ferritin tracer, ferritin standard and also used to produce the ferritin polyclonal antibodies.

### Ferritin extraction and purification from human spleen

After homogenation and heat denaturation step for 235 gm human spleen the extract undergo centrifugation and the protein content and ferritin content was measured in a sample from the 30 ml supernatant using commercial colorimetric kit (Spinreact) and enzyme immunoassay kit (Monobind) respectively, the results was 1306±4.19 mg and 24±0.18 mg respectively. The purity and recovery percent was calculated mathematically to be 1.84 % and 100% respectively.

After successive precipitation steps using 50% saturated ammonium sulphate and extensive dialysis the content of protein and ferritin in 25 ml crude extract was measured to be 480±1.54 mg and 22.3±0.175 mg respectively with purity and recovery percent to be 4.65 % and 92.9 % respectively

After dialysis, the crude ferritin extract was applied onto 16x90 cm gel filtration Sephacryl S-300 high resolution column, then eluted isocratically using 0.05 M phosphate buffer pH 7.4 and 5 ml fractions were collected at a flow rate of 0.5 ml/min. five Peaks was obtained each peak was estimated for ferritin content, the results show that the ferritin was concentrated in the second peak with protein concentration 60.5±0.475 mg and ferritin concentration 17.6±0138 mg with purity and recovery percent to be 29.09 and 73.3 % respectively

**Fig. 1:**
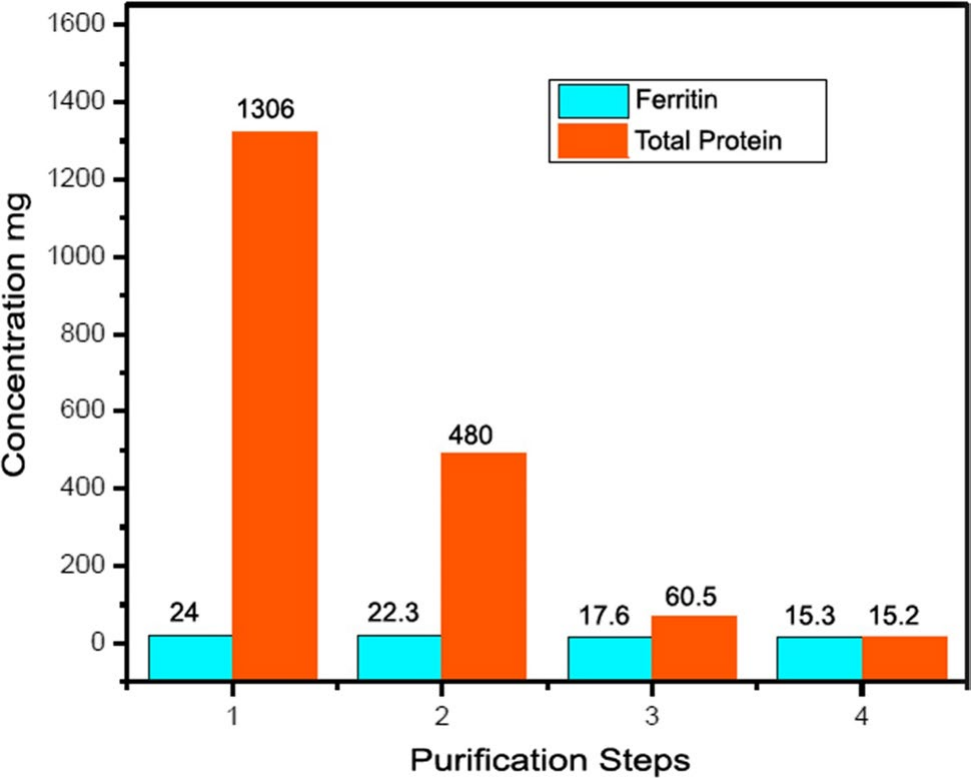
Ratio between ferritin and total protein during purification steps

The second peak was collected and reapplied on the same column for further purification. One sharp peak of highly purified ferritin was obtained (5 ml) and the protein and ferritin content was estimated, the results were 15.3±0.12 mg and 15.2±0.119 mg respectively**,** The purity and recovery percent was calculated mathematically to be 99.35 % and 63.3 % respectively as shown in Table 1 and the ratio between Ferritin and Total protein concentration during purification steps can be also summarized as shown in Figure 1

**Table 1:** Results of purification steps for human spleen ferritin

No	Purification step	Volume (ml)	Total protein (mg)	Total ferritin (mg)	Ferritin / protein ratio	Purity %	Recovery %
1	After heating the extract	30	1306 ± 4.19	24 ± 0.18	0.02	1.84	100
2	After Amm. Sulphate Precipitate	25	480 ± 1.54	22.3 ± 0.175	0.05	4.65	92.9
3	After 1st Sephacryl S-300 purification	10	60.5 ± 0.475	17.6 ± 0.138	0.29	29.09	73.3
4	After 2nd Sephacryl S300 purification	5	15.3 ± 0.12	15.2 ± 0.119	0.99	99.35	63.3

### The ^125^I- ferritin Tracer

Purification of ferritin tracer from the radioiodinated reaction mixture takes place on sephadex G-25 column and radiochemical yield and purity was calculated.

#### Radiochemical yield %

**Fig. 2:**
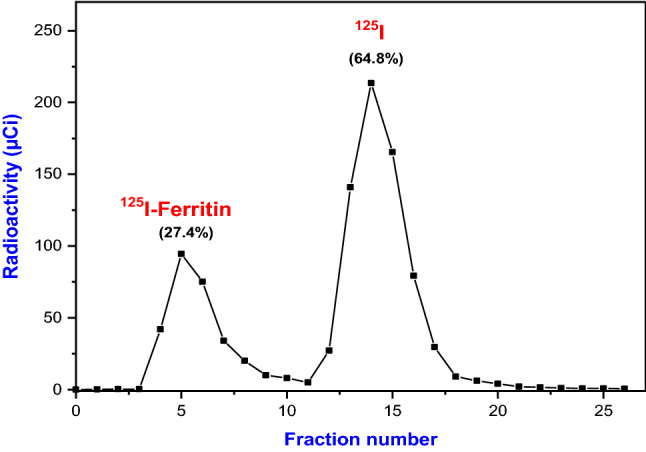
Purification of ^125^I- ferritin RIA tracer using sephadex G-25 column (flow rate 0.5 ml / min.)

The radiochemical yield percent calculated using the following equation:

${\text{Radiochemical~yield~\% }} = {\text{~}}\frac{{{\text{Radioactivity~of~}}{}_{{}}^{{125}} {\text{I}} - {\text{ferritin~tracer~peak~}}}}{{{\text{Total~radioactivity~of~fractions}}}} \times 100$The radiochemical yield percent of ^125^I-ferritin tracer was 27.4 ± 0.21% Fig. 2.

#### Radiochemical purity %

**Fig. 3:**
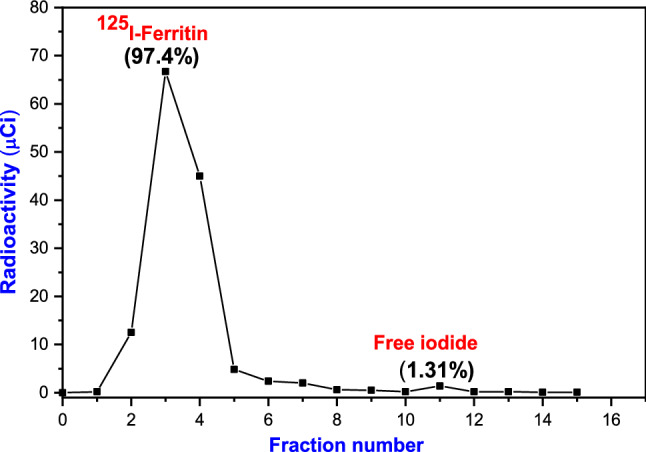
Electrophoretical pattern of radiochemical purity of produced ^125^I-Ferritin tracer

The purification profile of ^125^I-ferritn tracer was determined using paper electrophoresis and calculated as following:

${\text{Radiochemical~purity~\% }} = {\text{~}}\frac{{{\text{Radioactivity~of~}}{}_{{}}^{{125}} {\text{I}} - {\text{ferritin~tracer~peak~}}}}{{{\text{Total~radioactivity~on~the~paper~}}}} \times 100$It shows radiochemical purity percent of 97.4% as illustrated in Fig. 3 with specific activity of 26.5 ± 0.01 µCi/µg.

## Optimization of factors affecting the iodination process

**Fig. 4:**
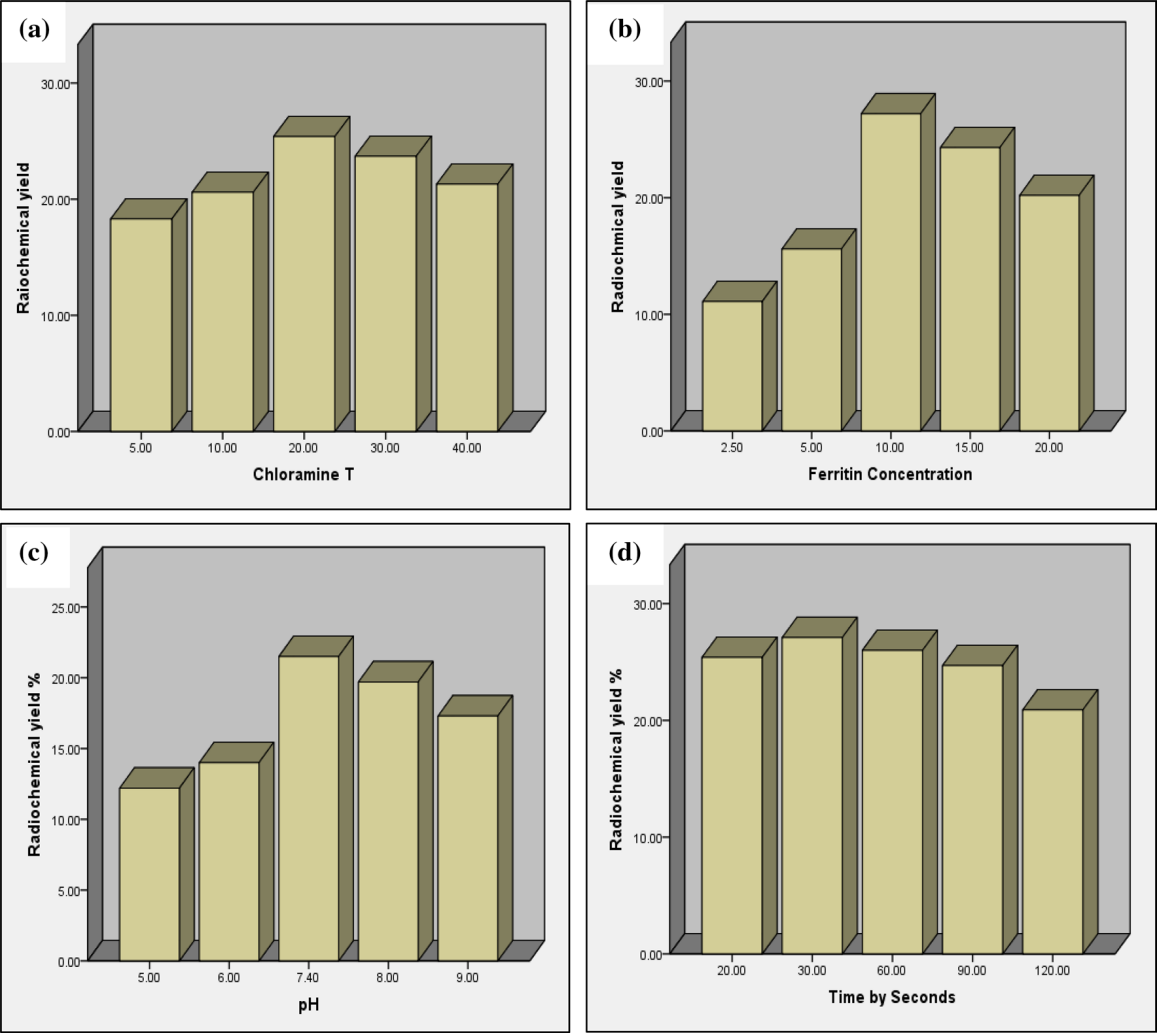
Factors affecting the radiochemical yield of ferritin tracera) Effect of chloramine-T concentration **b** Effect of ferritin concentration **c** Effect of pH **d** Effect of reaction time

Studying the factors affecting the radiochemical yield of ferritin tracer shows that:

The best radiochemical yield resulted when using 20 μg of chloramine-T and 10 μg of substrate (ferritin) at pH 7.4 for 30 s reaction time Fig. 4.

### Ferritin polyclonal antibody production

**Fig. 5:**
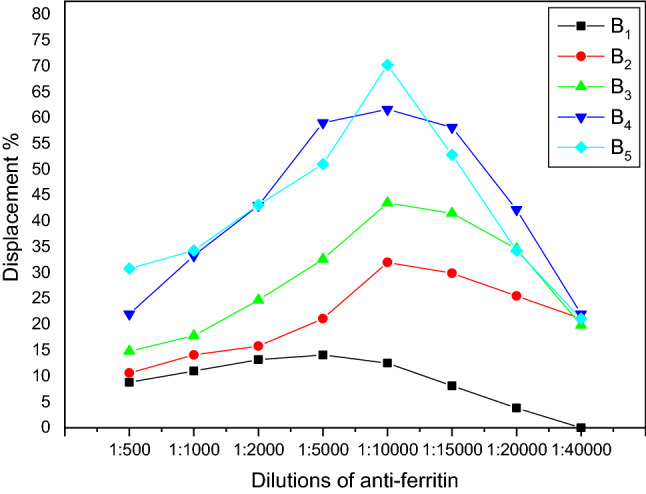
Displacement of ferritin Polyclonal antibody for rabbit no. 1 during the 5 bleeds

The data obtained from rabbits used in this study showed that the anti-sera obtained from bleed number five of rabbit no.1 gave the highest displacement percent 70.2 at dilution 1:10,000 as shown in Fig. 5.

### Ferritin radioimmunoassay optimum conditions

Optimum conditions for all parameters needed in radioimmunoassay system were studied to achieve the best results and accurate standard curve. The results can be summarized as following Table 2

**Table 2:** Optimum conditions used to construct the standard curve

Incubation time	Incubation temperature	Sample volume
3 h	37 °C	100 µl
PEG 12% volume	2nd antibody dilution	Normal rabbit serum dilution
500 µl	1: 40	1:200

### The optimized assay design

**Fig. 6:**
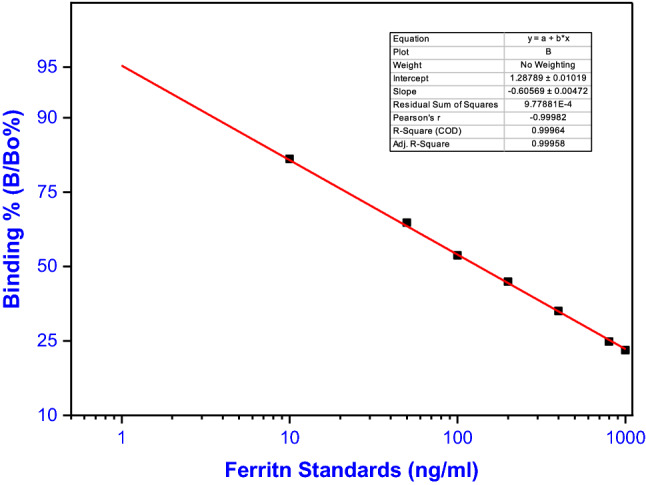
Optimized standard curve for ferritin using liquid phase RIA system

The assay was designed using optimum conditions studied as following: 100 µl ferritin standards or serum samples and 100 µl of locally prepared ^125^I-ferritin tracer were added to 100 µl ferritin polyclonal antibody (1/10,000 dilution of 5th bleed from rabbit 1) in polystyrene tubes. The contents of the tubes were mixed together and incubated for 3 h at 37 °C. The separating agent was introduced at the conclusion of the incubation period, commencing with 100 µl goat anti-rabbit IgG, 100 µl non-immunized rabbit serum, and 500 µl PEG-8000. Assay tubes were mixed with a vortex and incubated for 30 min at room temperature before being centrifuged at 4000 rpm for 20 min at 4 °C. The tubes were carefully decanted and precipitated antigen–antibody complex was counted in a multi-crystal gamma counter, the data were calculated, and the standard curve was drawn as shown in Fig. 6.

### Validation of the assay

To assure the validity and reliability of the suggested assay, some performance characteristics were studied.

#### Sensitivity of the assay

The results of the present study showed that the minimum detection limit of the assay is approximately 1 ng/ml which reflects the high sensitivity of this technique in measurement of ferritin in human serum Table 3

**Table 3:** The sensitivity of ferritin liquid phase RIA system

Cpm (Mean-SD)	Cpm (Mean-2SD)	B/Bo %	Apparent concentration (ng/ml)	Approximate sensitivity (ng/ml)
12,228–135	11,958	94.95	1	1

#### Precision

*Intra-assay precision (within run)*

Mean, Standard deviation and coefficient of variation were calculated for 10 replicates of low, medium and high human ferritin samples in a single run. The CV% results ranged from 1.7 to 4.5% Table 4.

**Table 4:** Intra-assay precision for ferritin using liquid phase RIA system

Samples	Intra-assay		
	Mean (ng/ml)	SD (ng/ml)	CV%
Low	14.6	± 0.65	4.5
Normal	122.5	± 2.1	1.7
High	346.1	± 6.3	1.8

*Inter-assay precession (Run to run)*

Mean, Standard deviation and coefficient of variation were calculated for 10 replicates of low, medium and high human ferritin samples in 10 separate runs. The CV% results ranged from 2.6 to 5.2% Table 5.

**Table 5:** Inter-assay precision for ferritin using liquid phase RIA system

Samples	Inter-assay		
	Mean (ng/ml)	SD (ng/ml)	CV%
Low	15.3	± 0.8	5.2
Normal	124.1	± 4.5	3.6
High	344.2	± 8.9	2.6

The present study's intra-assay and inter-assay data demonstrated that the results produced by the current procedure were consistent. The findings are in accordance with Pillai and Bhandarkar [21] and Ragab et al. [22], who stated that intra-assay CVs should be less than 10% and inter-assay CVs should be less than 15%.

## Accuracy

### Recovery test

As shown from results listed in Table 6, it can be observed that recovery percentage for ferritin samples (25.2, 185.6 and 412.5) ranged from (92.6 to 105.9), (92.7 to 104.3) and (89.49 to 107.8) respectively. The recovery data of the present study for ferritin indicates accurate calibration and an appropriate matrix.

**Table 6:** Recovery assessment for ferritin using liquid phase RIA system

Sample	Endogenous (ng/ml)	Added (ng/ml)	Expected (ng/ml)	Observed(ng/ml)	Recovery (%)
1	25.2	20	22.60	22.22	98.3
		150	87.60	92.77	105.9
		350	187.60	173.72	92.6
2	185.6	20	102.80	107.22	104.3
		150	167.80	155.55	92.7
		350	267.80	256.28	95.7
3	412.5	20	216.25	207.21	95.82
		150	281.25	251.69	89.49
		350	381.25	410.99	107.8

### Dilution test:

The results in Table 7 reveal the concentrations of three human samples (185.6, 412.5, 649.2) undiluted and at various dilutions in the assay buffer to assess the linearity of the assay. The recovery percentage was ranged from (94.7 to 102.3%), (93.6 to 105.3%) and (93.5 to 109.8%) respectively, this indicates to good linearity under several dilutions.

**Table 7:** Dilution test for ferritin using liquid phase RIA system

Sample	Endogenous(ng/ml)	Dilutionfactor	Expected(ng/ml)	Observed(ng/ml)	Recovery(%)
1	185.6	1:2	92.8	90.8	97.8
		1:4	46.4	47.5	102.3
		1:8	23.2	22.1	95.2
		1:16	11.6	11.0	94.7
2	412.5	1:2	206.3	209.3	101.5
		1:4	103.1	108.6	105.3
		1:8	51.6	48.3	93.6
		1:16	25.8	25.0	97.1
3	649.2	1:2	324.6	303.5	93.5
		1:4	162.3	160.8	99.1
		1:8	81.2	83.2	102.5
		1:16	40.6	44.6	109.8

#### Method comparison

**Fig. 7:**
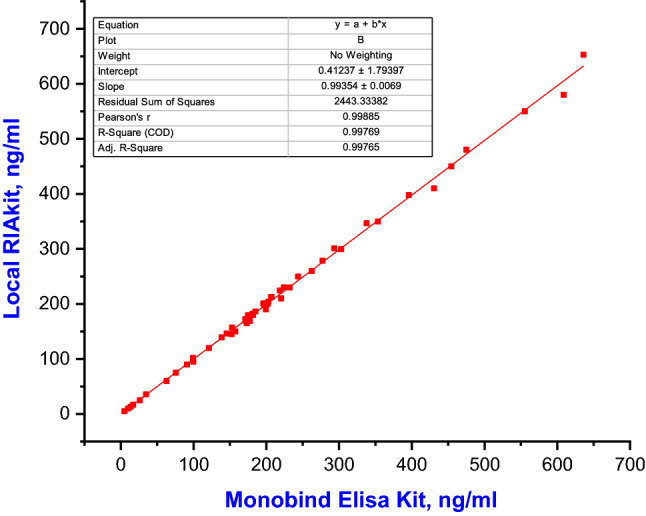
Regression line equation and correlation coefficient "r" between ferritin values obtained by Monobind Elisa kit and the local liquid phase RIA system

The statistical analysis (linear regression and correlation coefficient "r") were carried out to compare ferritin results for 50 different human serum samples with values ranged from 5 to 650 ng/ml measured by the prepared system (ferritin liquid phase RIA) versus results measured using commercially available kits (Monobind Elisa kit).The statistical calculations show good correlations between the results obtained from the prepared system and the commercially available kits (r = 0.998) Fig. 7.

## Conclusion

Radioimmunoassay system for ferritin in this report was developed, optimized and validated. The assay exhibited high senstivity of 1 ng/mL, acceptable imprecision level of less than 5.2%, recovery between 89.49 and 107.8% and linearity of dilution between 93.5 and 109.8%. The developed assay system can be used for routine sample analysis of ferritin in human serum as a laboratory biomarker in Covid-19 patients.
